# Health Benefits of Apple Juice Consumption: A Review of Interventional Trials on Humans

**DOI:** 10.3390/nu14040821

**Published:** 2022-02-16

**Authors:** Bastien Vallée Marcotte, Marie Verheyde, Sonia Pomerleau, Alain Doyen, Charles Couillard

**Affiliations:** 1 Institut sur la Nutrition et les Aliments Fonctionnels (INAF), Université Laval, Québec, QC G1V 0A6, Canada; bastien.vallee-marcotte.1@ulaval.ca (B.V.M.); marie.verheyde.1@ulaval.ca (M.V.); sonia.pomerleau@fsaa.ulaval.ca (S.P.); alain.doyen@fsaa.ulaval.ca (A.D.); 2 Centre Nutrition, Santé et Société (NUTRISS), Université Laval, Québec, QC G1V 0A6, Canada

**Keywords:** apple juice, interventional trials, health benefits

## Abstract

Although numerous studies have reported the benefits of apple consumption on cardiometabolic health parameters and chronic disease prevention, few have focused on the effects of apple juice specifically. Juice consumption may be a convenient way to take advantage of the health effects of the bioactive components present in apples. The present review aims to summarize the current literature on health benefits of apple juice as reported in clinical trials in humans. Of the 67 studies retained, 20 interventional studies on humans were reviewed. Overall, cloudy apple juice consumption was found to be associated with several markers of cardiovascular health that may ultimately be relevant for cancer and neurodegenerative diseases. Most of the documentation was found regarding oxidative stress, as well as observations with other parameters such as markers of inflammation, lipid profile, and diabetes. This review suggests that, in 20 studies, apple juice consumed in moderation exerts positive effects on markers of cardiovascular disease risk (particularly on oxidative stress).

## 1. Introduction

Data from the scientific literature supporting that fruits and vegetables (FAV) consumption is beneficial for cardiovascular disease prevention are quite undisputed. However, the health benefits of FAV extend beyond their cardioprotective effects. Many biologically active components in FAV can play a preventing role in the development of a various range of chronic diseases including cancer and neurodegenerative diseases, for which many studies have investigated the effect of different fruit supplementations on related markers such as Wnt signaling, mucosal proliferation [1] and the neuronal response to working memory load conditions [2]. The protective effect of FAV consumption is likely due to their high content in nutrients, fibers, and phytochemical components, as well as their low energetic density [3]. Since fruits do not have the same nutritional or energetic density, they are likely to have inequivalent properties, and thus it becomes relevant to investigate the contribution of specific fruits to disease prevention.

Apples are a good example of fruit that has caught the attention of researchers for several reasons. Besides being very accessible, versatile, and inexpensive, apples have a high nutritional value and contain a wide variety of bioactive components, making them a fruit of interest. Although apple consumption has been associated with several positive health outcomes, fewer reports have documented whether apples consumed in different forms, like apple juice, pomace, cider, vinegar, and others, exert the same beneficial effects as whole fruits.

Some experts affirm that whole apples have superior nutrient density, while others point out that juice or liquid forms can be more convenient [4]. Indeed, a great advantage of juice is its ease of consumption, making it an interesting option, when taken with moderation, to take advantage of the health properties of fruits. It has been reported that individuals consuming fresh fruit juice are closer to meeting the daily recommendations for fruit intakes [5]. A study based on data from the 2013–2016 National Health and Nutrition Examination Survey reported that 100% fruit juice consumers had a 10% higher Healthy Eating Index 2015 score [6] compared to non-consumers as well as higher intakes of a variety of nutrients such as vitamins C and D, calcium, magnesium, potassium, and energy.

While epidemiologic as well as in vitro and animal studies have investigated the relationships between apple juice consumption and health, clinical trials have emerged in recent years as a way to provide evidence of great quality. The present review aimed to clarify the effects of apple juice consumption on health and disease prevention by summarizing the recent literature from interventional studies in humans.

## 2. Overview of Bioactive Components in Apples and Apple Juice

The literature is fairly consistent regarding the high nutritional value of apples. Indeed, apples are rich in many nutrients and contain several components that have been shown to have bioactive effects such as phytochemicals, vitamin C, dietary fibers, and pectin (Table S1) [7].

### 2.1. Phytochemicals

Many of the health benefits attributed to phytochemicals are due to their antioxidant activity. Antioxidants are molecules that are able to expend electrons to other, unstable, molecules called free radicals to prevent cellular damage [8]. This class of molecules includes carotenoids-such as vitamin A-vitamin C and E, selenium, and polyphenols among others [9,10]. Apple polyphenols have been shown to prevent lung tissue damage from smoking [11] through antioxidant effects and could decrease low density lipoprotein (LDL) oxidation [12,13]. By reducing oxidative stress, their actions could overall reduce aging-induced cell damage and play a role in the prevention of a wide variety of conditions, including type 2 diabetes, cancer, hypertension, asthma, infections, cardiovascular disease, neurodegenerative disease, and many others [14,15,16,17].

Phytochemicals present in apples are mostly polyphenols. Polyphenols are a subclass of phytochemicals. Five main polyphenols groups are present in apples, namely flavanols (catechins, epicatechin and procyanidins), flavonols (quercetin glycosides), phenolic acids (chlorogenic, gallic and coumaric acids), dihydrochalcones (phloretin glycosides), and anthocyanins (cyanidin) [16,18]. Concentrations of phytochemicals vary with cultivar, harvest and storage [16]. Polyphenols can be lost in part during juice processing mostly due to the oxidative conditions of pulping, pressing and clarification [19,20]. Accordingly, cloudy apple juice has a higher content of polyphenols compared to clear apple juice [19,20].

### 2.2. Vitamin C

Vitamin C is highly present in fruits and is a strong antioxidant not only capable of disabling free radicals but also implicated in the regeneration of vitamin E, protecting cells and molecules against oxidative damage which contributes to prevention of several diseases [21]. Vitamin C is also an electron donor for enzymatic and non-enzymatic functions [21] and a co-factor for ferrous and 2-oxoglutarate dependent dioxygenases, two enzymes implicated in collagen synthesis through hydroxylation of proline and lysine [22]. It also acts as a co-factor for enzymes catalyzing several biological processes including muscle carnitine and noradrenaline biosynthesis, amidation of peptide hormones, iron homeostasis through hypoxia-inducible factor hydroxylation, histone demethylation, and tyrosine metabolism [23]. Vitamin C deficiency leads to impairment of wound healing and bleeding, and eventually scurvy [22] which occurs as a result of prolongated avitaminosis [21]. Finally, vitamin C improves vascular function by increasing nitric oxide release, facilitating vasodilatation through relaxation of smooth muscle cell [21]. Low levels of vitamin C has also been linked to increased risk of cardiovascular disease [23,24]. Vitamin C in juice is relatively unstable and is very sensitive to heat, oxygen, pH and light [25] which explains why whole fruits generally have higher vitamin C content than their juice.

### 2.3. Fibers

Fibers are non-digestible polysaccharides that can exert positive health benefits through their passage into the gastro-intestinal tract [26]. There is a strong relationship between consumption of dietary fibers and risk reduction of several diseases and disorders, including cardiovascular disease, diabetes, obesity, gastrointestinal disorders, and cancers [27]. Foods with a high fiber content have high satiating power, which helps to decrease food and energy intake at mealtimes [28,29]. Fibers can be classified into two main groups: soluble and insoluble fibers [26]. Soluble fibers act as a sponge by jellifying in water and can create chemical bonds between molecules, whereas insoluble fibers have more properties that are more physical [26,30]. In the small intestine, fibers that binds bile acids and increases excretion of lipids in the feces [29,31]. By delaying macronutrients absorption, they also attenuate postprandial glucose and insulin increases [31]. In the colon, soluble fibers are fermented by bacteria to produce biologically active short-chained fatty acids (SCFA) that are absorbed in the system and contribute to decrease hepatic cholesterol synthesis [27,31]. Fibers also provide stimulus to the bowel that helps preventing intestinal disorders such as diverticular disease and hemorrhoids, constipation, and diarrhea among others [31]. Regarding cancer prevention, several studies have shown that high fiber intake could prevent colorectal cancer [31].

### 2.4. Pectin

Pectin is a polysaccharide from plant cell wall which in addition to possessing the properties of fibers [32,33,34,35] also has an antioxidant capacity which could prevent the development of cancers [31]. One particularity of pectin is its high solubility in water gel forming ability in the small intestine, which makes it a powerful hypoglycemic agent [36,37,38]. Due to its viscosity, pectin can also bind to several metals facilitating their elimination while also reducing the absorption of toxic metals [38] such as arsenic, cadmium, lead, and mercury which have been shown to disturb endocrine, nervous and immune systems [38]. Regarding potential anticarcinogenic properties, pectin could play a preventive role in a wide variety of cancers, including prostate, pancreas, breast and colorectal cancer, and could even exert chemoprotective effects on metastasis [38]. More specifically, pectin could have antiproliferative actions by inhibiting cell proliferation, migration, adhesion, and triggering of apoptosis [38]. Finally, it exerts prebiotic effects in the colon since it is highly fermentescible [38]. Several studies showed that pectin, and even apple-derived pectin, could exert beneficial effects on health through modulation of gut microbiota, and could decrease inflammation and the presence of endotoxins [34,39,40]. As with other dietary fibers, pectin is almost completely removed from juice during filtering.

### 2.5. Nutritional Value of Fruit Juices and Health

Typically, ready-to-drink apple juice is obtained from several processing steps including washing, milling, and pressing to optimize juice extraction [41,42,43]. Since apple juice is mainly consumed as a brightly clear product, a clarification step of the raw apple juice is necessary to remove suspended solids and pectin. The clarification step is conventionally achieved by addition of filtering agents (gelatin and bentonite) and/or pectic enzymes [41,42,43,44]. Particles in suspension are then removed by centrifugation or pressure-driven filtration processes [41,42,43,44]. Finally, a thermal treatment (pasteurization or sterilization) is applied on the clarified apple juice to improve its shelf-life [43,44]. These different processing steps have significant impact on health-promoting phytochemicals in apples. It was demonstrated that oxidation of phenolic compounds occurred during milling and pressing [44]. Moreover, a loss of phenolic compounds was observed after conventional clarification steps using gelatin and bentonite as well as filtration [41,42]. The loss of vitamin C, a thermosensitive compound, is mainly caused by the application of thermal treatment (pasteurization and sterilization) [45,46,47]. However, flash pasteurization is considered as an efficient and gentle food processing to better preserve vitamin C compared to conventional thermal technologies [45]. It is important to mention that the loss of bioactive compounds is largely correlated to the severity of processing parameters. Regarding dietary fibers initially present in apples, they are totally absent in the clarified apple juice since they are mainly concentrated in the apple pomace (apple juice by-products) [48] and eliminated during treatments by pectic enzyme and filtration [32,49,50,51].

Despite the negative impact of food processing on health-promoting compounds of apples, currently conflicting results are existing regarding the nutritional value of fruit juice (Table S2) [7,43]. On one hand, previous research reported that the nutritional value of fruit juice is quite similar to whole fruits, with the exception of fibers and vitamin C [52]. Despite that apple juice has depleted contents in phenolic compounds compared to whole apples, an interventional study published by Wruss et al. on humans demonstrated that consumption of unfiltered apple juice increased concentrations of phenolic compounds in blood and urine, with a high inter-individual variability between subjects [53].

On the other hand, some studies point out that the juicing process results in a too big loss of in nutrients for juice to be considered an acceptable substitute to whole fruits. For instance, one interventional study concluded that dietary fibers were necessary to induce the cholesterol-lowering effects of apples, and that clear apple juice was not an equivalent replacement to whole apples in terms of nutritional value [32]. As aforementioned, apple juice content in polyphenol and vitamin C is also reduced following processing and juicing, and fibers are almost completely absent from clear apple juice [32,46,47]. Considering that apple juice encounters major losses in nutrients during processing, it is likely that the beneficial effects of apple juice is attributable in great part to its content in soluble and most stable compounds such as phytochemicals.

## 3. Cardiovascular Disease and Associated Risk Factors

To conduct the present review, we searched the PubMed database and Google Scholar for interventional trials on humans. Trials had to include at least one of the following interventions: 1- single dose or supplementation with natural clear apple juice; 2- single dose or supplementation with commercial cloudy apple juice; and/or 3- single dose or supplementation with homemade juice from ground apple. Trials including only artificial juice, juice from fruit blends, whole apples, apple cider, apple vinegar, and/or apple pomace (the remaining solid part of the fruit after juice extraction) were excluded. Participants did not have to be healthy.

Trials conducted on animal models were excluded. Trials had to provide an assessment of the impact of interventions on biomarkers or a health outcome. Keywords used included Fruit and Vegetable Juice, Juice, Extract, Fruit Juice, Malus, Apple, Apple juice, Apple fibers, and/or Cloudy Apple Juice; and Cardiovascular Diseases, Diabetes, Glucose, HOMA, Insulin, Homeostasis, Lipids, Cholesterol, Triglycerides, Inflammation, Oxidation, Oxidative Stress, TEAC, ORAC, FRAP, Antioxidants, Antioxidant Capacity, Risk factors, Biomarkers, Obesity, Body Weight, Weight Loss, Body Mass Index, Fat, Cancer, Tumor, Gut, Microbiota, Microbiome, Neurodegenerative Disease, Cognitive, Brain, Alzheimer’s Disease, Parkinson Disease, Neurodegenerative, and Health. A summary of included interventional trials is presented in Table 1.

### 3.1. Diabetes

A few studies investigated several parameters of glucose homeostasis in interventions on humans using either apple juice supplementations or single doses of apple juice.

One of the most important studies on the subject was conducted by Ravn-Haren et al. [32] and addressed the effects of apple juice consumption on a variety of risk factors of cardiovascular disease. More specifically, it was a randomized, single-blinded, crossover study of 5 × 4 weeks that aimed to investigate whether consumption of whole apples or apple derivatives would differentially affect common risk factors of cardiovascular disease than processed apple fractions. The five interventions were: (1) control (restricted diet with no supplementation) or restricted diet with either (2) whole apple supplementation; (3) apple pomace supplementation; (4) cloudy apple juice supplementation; and (5) clear apple juice supplementation [32]. A total of 23 subjects completed all five interventions [32]. Authors reported no effect of any supplementations on insulin, insulin-like growth factor and Insulin-like growth factor-binding protein 3 concentrations [32]. The effect of supplementations on glucose levels were not tested. However, the effects on triglycerides were [32]. It is well-documented that triglyceride levels are associated with glucose levels and blood levels of both markers substantially increase in the post-prandial phase [49,54]. No effect on triglycerides levels was observed [32].

In another randomized interventional controlled trial by White et al. [50], in which participants received single doses of either apples, 100% apple juice, or control (fructose or glucose beverage), blood glucose was normally elevated 30 min following the intake and reflected the amount of glucose in the food for all interventions. Surprisingly, blood glucose was not elevated and even tended to decrease after two hours following 1 L single-dose consumption of either clear apple juice, clear apple juice supplemented in polyphenols and cloudy apple juice [55]. These results were also observed in a study by Godycki-Cwirko et al. [55], in which 12 healthy men consumed three times in four days either 1 L of clear apple juice with no polyphenol (control), 1 L of cloudy apple juice (positive control), or 1 L of water (negative control). Vieira et al. [51] reported similar observations in a study in which consumption of 300 mL of fresh apple juice made from peeled ground apples was no associated with an elevation of plasma glucose levels. In their study [51], nine healthy women consumed a 300 mL single dose of either tap water (control), Golden Delicious apple juice or Catarina apple juice, with washouts periods of two weeks in between. Blood samples were taken before and after the interventions [51].

In another study by Soriano–Maldonado et al. [56], participants consumed either cloudy apple juice rich in vitamin C or cloudy apple juice rich in polyphenols on a daily basis during four weeks and had increased homeostatic model assessment (HOMA) index and insulin levels following supplementation with the polyphenol-rich apple juice. A trend towards decreased triglyceride levels at post-intervention time was also observed in those who consumed the polyphenol-rich juice [56].

### 3.2. Lipid Profile

In the study aforementioned by Ravn–Haren et al. [32], cloudy apple juice and clear juice differentially affected total cholesterol (TC) and low-density lipoprotein cholesterol (LDL-C) levels. TC and LDL-C were significantly increased following clear apple juice intervention in comparison to apple and apple pomace interventions, in which a decrease was observed. TC and LDL-C were non-significantly decreased following cloudy apple juice intervention [32]. However, women showed some negative effects after clear apple juice consumption [32], more specifically increased LDL-C levels, whereas this effect was not significant in men [32]. There was also a difference on LDL-C between cloudy apple juice and whole apple and apple pomace in the women group [32]. No effect on HDL-cholesterol (HDL-C) or triglycerides was observed [32].

In another non-blinded, randomized, six-week crossover study, by Hyson et al. [57], healthy participants were provided 375 mL of unsweetened, unsupplemented apple juice or 340 g of apple flesh per day during six weeks. No significant change in total cholesterol, LDL-C, HDL-C, apolipoprotein AI or B levels. Plasma triglycerides were slightly elevated following interventions, but the association was not significant [57]. Similar inconclusive results regarding the effect of cloudy apple juice supplementation on lipid profile were also reported by Barth et al. and Soriano-Maldonado et al. in studies with similar design [56,58].

### 3.3. Inflammation

Effect of apple juice supplementations on inflammation markers were assessed in a few studies, often using markers like high-sensitivity C-reactive protein, adipokines, and other markers of vascular inflammation, with inconclusive results [32,58]. Barth et al. [58] found a gene-diet interaction between cloudy apple juice supplementation and one single nucleotide polymorphism in the IL-6 gene (IL-6-174 G/C) on body fat percentage. The study was a randomized controlled trial in which 68 men completed a four-week supplementation of 750 mL a day of polyphenol-rich cloudy apple juice [58]. A significant reduction in body fat percentage was only observed in homozygous participants carrying the mutated allele C [58].

In the study by Soriano-Maldonado et al. [56], in which participants consumed cloudy apple juice rich in vitamin C or cloudy apple juice rich in polyphenols daily for four weeks, the anti-inflammatory potential of daily supplementation with cloudy apple juice rich in vitamin C and cloudy apple juice rich in polyphenols was assessed using interleukins and adhesion molecules as markers in the plasma. They found no effect of supplementations on studied markers. However, there were significant differences in the between-treatment change from pre- to post-intervention for two adhesion molecules, namely intercellular adhesion molecule 1 and vascular cell adhesion protein 1 [56].

### 3.4. Oxidative Stress and Antioxidant Capacity

Many interventional studies addressed the effect of apple juice consumption on oxidative stress and antioxidant capacity. In the study by Ravn–Haren et al. [32], activity of erythrocytes antioxidant enzymes, namely glutathione peroxidase, glutathione reductase and catalase, was assessed following supplementations of clear and cloudy apple juices, apple pomace and whole apples. Most interventions, including cloudy apple juice and clear apple juice, significantly decreased the activity of glutathione peroxidase. However, no effect on other antioxidant enzymes was observed [32]. Antioxidant activity was measured in the plasma using three methods, ferric reducing ability of plasma (FRAP), Trolox equivalent antioxidant capacity (TEAC), and oxygen radical absorbance capacity (ORAC). FRAP is a test that measures the ferric to ferrous reduction ability of plasma for assessing the antioxidant capacity [59]. TEAC uses the radical 2,2’-azinobis-(3-ethylbenzothiazoline-6-sulfonic acid) to assess antioxidant capacity and is commonly used for building structure activity relationships [60]. ORAC is a test used to quantify the oxygen-radical absorbing capacity of antioxidants in serum by completing the oxidation process [61]. ORAC was significantly increased with cloudy apple juice compared to control [32]. A trend towards increased FRAP was observed with cloudy apple juice, while FRAP was non-significantly decreased with clear apple juice. No effect was found on TEAC [32]. Plasma lipid resistance to oxidation was also assessed, and was almost significantly associated with overall dietary interventions [32].

Similar findings were reported by Wruss et al. [53] in an interventional study that aimed to test whether consumption of polyphenols contained in apple juice had an impact on antioxidant capacity in the plasma. Blood and urine samples were collected several times following ingestion of a unique dose of 500 mL of unfiltered apple juice and antioxidant capacity was assessed using TEAC and ORAC [53]. ORAC highly increased in blood one hour following apple juice consumption, then dropped significantly in the following hours before increasing again six hours after [53]. This observation was confirmed by TEAC. However, total phenolic content and phenolic content analysis by reverse phase chromatography in urine did not match this kinetic pattern [53]. In another study by Godycki-Cwirko [55], participants consumed, on three separate occasions, single dose of either 1 L of clear apple juice without polyphenols, clear apple juice with polyphenols or cloudy apple juice. FRAP and serum 2,2-diphenyl-1-picrylhydrazyl radical-scavenging activity were increased following consumption of all three juices and, similarly to results from other studies, peaked after one hour [55]. FRAP was still elevated 2.5 and 4 h following consumption of cloudy juice and clear juice without polyphenols [55]. A positive correlation between FRAP and uric acid concentrations was observed [55]. No change in plasma polyphenols and quercetin levels were observed following interventions [55]. Overall, all three juices had a similar effect on studied parameters [55]. FRAP and 2,2′-azinobis (3-ethylbenzothiazoline-6-sulfonic acid) (ABTS), another measure of antioxidant capacity, were also increased following consumption of 300 mL of fresh apple juice in a similar study by Vieira et al. [51]. In addition, authors reported a significant decrease in lipid peroxidation, more specifically in lipid hydroperoxides and thiobarbituric acid reactive substances (TBARS) [51]. Soriano-Maldonado et al. [56] also observed a significant reduction in FRAP following supplementation of eight men and 12 women with cloudy apple juice rich in vitamin C. More specifically, participants consumed 500 mL per day of either a polyphenol-rich or vitamin-C rich cloudy apple juice for four weeks in a cross-over design, with a washout period of two weeks in between [56]. No reduction was observed with the supplementation of cloudy apple juice rich in polyphenols. On the other hand, the cloudy apple juice supplementation that was rich in vitamin C induced a significant decrease in total glutathione, but not the polyphenol-rich one [56].

Another study in which ten healthy men consumed a single dose of 150 mL of apple juice from ground apple flesh reported an increase in antioxidant capacity in the blood, evaluated by reactive oxygen species inhibition using dichlorofluorescein fluorescence [62]. The study was a cross-over trial in which participants drank a single dose of juice made from ground fruit (pear, apple, orange, grape, peach, plum, kiwi, melon, and watermelon), with one-day washouts in between [62]. Following the apple juice dose, antioxidant capacity peaked 30 min after consumption and remained stable until 1.5 h after consumption [62]. In another study, total plasma antioxidant capacity, as well as glutathione peroxidase and catalase activities, were improved in healthy subjects following a two-week supplementation of 300 mL apple juice and 300 mL of grape juice every day [63]. It is unclear, however, whether the observed effects were driven by one of the two juices.

These findings are consistent with observations reported by Guo et al. [64], who conducted a study that aimed to compare the effects of pomegranate juice with apple juice on antioxidant function in elderly participants. Their reports showed an increased antioxidant capacity following supplementations of either 250 mL of pomegranate or apple juice for four weeks, with a more pronounced impact following the pomegranate intervention [64]. More specifically, apple juice consumption induced an increase in plasma glutathione peroxidase and catalase activities, and a decrease in malondialdehyde, a peroxidized fatty acid [64]. In the aforementioned study by Hyson et al. [57], apple juice supplementation significantly decreased susceptibility of LDL to oxidation (increased lag time) and decreased the overall production of peroxidized lipids. In this study, healthy participants followed a supplementation of 375 mL of unsweetened, unsupplemented apple juice or 340 g of apple flesh per day during six weeks [57].

### 3.5. Microbiome and Intestinal Health

In recent years, there has been an important increase in the interest to study the microbiome as a modulator of the cardiometabolic risk profile [65,66]. The emerging science of microbiome has even started transitioning from fundamental research to clinical practice [65,66]. Clinical studies linking the microbiome to apple juice have also been conducted.

Ravn–Haren et al. [32] investigated whether apple or apple derivatives supplementations affected composition of the gut microbiota using denaturing gradient gel electrophoresis. Their study was a randomized, single-blinded, crossover study of 5x4 weeks consisting of five supplementations (control, whole apples, apple pomace, cloudy apple juice and clear apple juice) [32]. No apparent change was observed on the gel [32]. Besides, other research groups found in interventional studies on patients with ileostomy that consumption of cloudy apple juice or apple smoothie containing apple juice could increase the polyphenol content of ileostomy bags, mainly about two hours post-consumption, thus suggesting that polyphenols could reach the colon in healthy subjects and exert antioxidant activity in the lower intestinal tract [67,68,69]. As mentioned by Hagl et al. [67], such findings might show that apple products such as apple juice could play a preventive role in the development of colon diseases by increasing the colonic availability of polyphenols.

Moreover, Trošt et al. characterized metabotypes in response to cloudy apple juice enriched or not with polyphenols and found correlations between the composition of the gut microbiota and patterns of microbial catabolites derived from apple polyphenols that were observed in urine and plasma following consumption of different apple juices [70]. Authors concluded that various members of the gut microbiota could act together in the metabolization of polyphenols from plant-based food [70]. These overall findings clearly support that components in apple juice interact with the gut microbiota to exert potential biological activity [70]. Finally, one study reported that apple juice was well tolerated regarding gastrointestinal comfort despite its high content in fermentable oligosaccharides, disaccharides, monosaccharides and polyols (FODMAP) [71].

### 3.6. Obesity

No effect of apple juice on body weight was observed in the interventional study by Ravn–Haren et al. [32]. Nonetheless, Barth et al. [58] had male participants supplemented with 750 mL/day of cloudy apple juice for four weeks and observed a slight but significant decrease in body fat percentage after the intervention. As previously mentioned, a gene-diet interaction was observed between cloudy apple juice supplementation and one single nucleotide polymorphism in the INTERLEUKIN-6 gene on body fat percentage [58]. Homozygous carriers of the minor allele of the IL-6-174 G/C polymorphism had decreased body fat percentage following the intervention as opposed to non-carriers and heterozygous, for whom the reduction was not significant [58]. Again, no association was found with body mass index and waist circumference [58].

### 3.7. Other Risk Factors of Cardiovascular Disease

In the randomized controlled trial by White et al. [50], authors observed that apple juice consumption had no impact on blood pressure. The primary objective of this trial was to compare the acute effects of fructose consumption between whole apples vs. apple juice on uric acid levels, a marker increased with fructose metabolization by the liver that has been identified as a risk factor for hyperuricemia [50]. They observed a similar response of uric acid levels following fructose consumption regardless of the source in a dose-dependent relationship [50]. Consistent findings were reported by Godycki–Cwirko, where uric acid was found to increase and peaked one hour following a single-dose consumption of 1 L of clear apple juice, clear apple juice supplemented in polyphenols and cloudy apple juice [55]. Uric acid was also increased one hour following consumption of 300 mL of fresh apple juice as previously reported by Vieira et al. [51].

## 4. Cancer

To our knowledge, only one interventional study on humans investigated the potential health benefits of apple juice consumption on markers of cancer. According to this pilot study by Veeriah et al. [69], in which patients with ileostomy ingested a single dose of 1 L of cloudy apple juice, cloudy apple juice could exert antigenotoxic effects in the intestine. In this study, HT29 cells were incubated with ileostomy samples and DNA damage was induced following preincubation using H_2_O_2_ to assess antigenotoxic capacity [69]. H_2_O_2_ was used as positive control for DNA damage as well [69]. A trend towards reduced genotoxic activity was observed in many subjects. However, a large inter-individual variability was observed in the response of H_2_O_2_-induced DNA damage of HT29 cells [69]. Authors explained that the increased content of polyphenols in the gut lumen following cloudy apple juice consumption could decrease the exposure to genotoxins and pro-oxidant agents [69].

Oxidative stress impairs the antioxidant defense of the body which may predispose to the development of cancer through DNA damage, genome instability and cell proliferation [72]. In this regard, studies that investigated the effect of apple juice consumption on markers of oxidative stress, even if for cardioprotective interests, may provide relevant information on the potential of apple juice to have an impact in cancer prevention. As indicated earlier, evidence from available studies reveal that apple juice consumption can reduce oxidative damage to lipids and potentially DNA and proteins, and that cloudy apple juice may have more potent effects than clear apple juice. Likewise, inflammation, obesity and microbiome dysbiosis have also been associated with cancer [73,74,75], and any benefits of these conditions in response to the consumption of apple juice may warrant further investigation.

## 5. Neuroprotection

Few clinical studies on the effect of apple juice consumption and cognitive health have been conducted in humans. Significant improvements in behavioral and psychological symptoms of dementia (anxiety, agitation, delusion) in individuals diagnosed with moderate-to-late stage of Alzheimer’s disease who consumed 237 mL (8 oz) of apple juice for one month [76]. However, no improvement in cognitive performance was observed in these individuals according to the Dementia Rating Scale 2 or daily living functioning according to the Alzheimer’s Disease Cooperative Study-Activities of Daily Living [76].

Lower hemoglobin status and anemia have been associated with increased risk of dementia and Alzheimer’s disease [77,78]. Consumption of apple juice was found to be as effective in increasing iron absorption at mealtime as orange juice in children, thus suggesting it could be a useful tool in the prevention of iron deficiency and anemia [79].

As for cancer and cardiovascular disease prevention, the beneficial impact of apple juice on oxidative stress, gut microbiota, could also be beneficial for preventing neurodegenerative disorders [80,81,82,83].

## 6. Discussion

In the present review, we aimed to assess the potential health benefits of apple juice consumption based on high quality evidence by gathering information strictly from interventional trials on humans. Overall, results from studies that were included in the present review suggest that consumption of apple juice could exert some benefits on a variety of markers associated with the risk of developing chronic diseases. Apple juice supplementations as well as single-dose apple juice consumption induced a response of several markers. Oxidative stress was the most studied aspect of the health effects related to apple juice consumption, which is expectable considering their high content in bioactive compounds, including phytochemicals, vitamin C, dietary fibers and pectin, and their strong antioxidant activity. Most documentation found abrupt response of antioxidant capacity within few hours following consumption. For some markers, such as lipid profile, the effect of apple juice appeared to have very modest effect. This variability might be attributable to the possibility that markers evaluated respond to different components in apple or apple juice. For instance, the beneficial effects of apple juice consumption on lipid peroxidation might be mainly attributable to polyphenols, which are very present in apple juice, and their antioxidant capacity, whereas the lipid lowering effect of apples might rather be induced by dietary fibers that are present in apples, but not in apple juice.

In most studies, cloudy apple juice appeared superior to clear apple juice regarding health benefits. Clear apple juice is more filtered than cloudy apple juice, and the latter is therefore much closer to the whole fruit. Clear apple juice has a lower content in dietary fibers, phytochemicals and other components that are present in the cell wall than clear apple juice [32,46,47].

Regarding energetic value, cloudy apple juice and clear apple juice contain similar amounts of sugar. It is important to underline that despite the potentially beneficial effects of apple juice, overconsumption of juice may lead to adverse health effects. Several studies concluded that excessive juice consumption may contribute to obesity and recommend to limit its consumption, mainly due to its high content in sugar [84,85,86]. Randomized-controlled trials also showed that 100% fruit juice could increase tooth erosion and dental caries [87]. However, moderate consumption of juice could be of great help for achieving recommended daily intakes of fruits and vegetables [85].

The present review focused on interventional trials on humans rather than epidemiologic research or in vitro and animal studies. The body of literature of non-interventional studies on the beneficial effects of fruit consumption [17], and more specifically apples and its juice are consistent with the results from interventional trials. These studies brought out a lot of associations between apples, apple juice, and other apple components and numerous conditions and disease risk factors such as cancer, antioxidant activity, cardiovascular disease, asthma, pulmonary function, cognitive function, bone health, body weight, glucose homeostasis, gastrointestinal function, microbiome, and others [17]. However, these observations have not always been confirmed or investigated in clinical studies (such as for lung function).

For instance, the relationship between asthma and apple juice consumption has been studied in epidemiologic studies. Okoko et al. investigated the potentially protective effects of apple juice consumption on childhood asthma in a population-based survey of 2640 elementary school children and found that drinking apple juice once a day was inversely associated with wheezing [88]. Surprisingly, no association was found with daily intake of whole apples [88]. However, to our knowledge, this relationship was never investigated in clinical trials on humans, although the impact of whole fruit consumption, including apples, on the risk of asthma and wheezing was investigated in many studies, and the overall body of literature trends towards a protective effect [89].

Other studies such as in vitro studies uncovered several potential mechanisms explaining associations found in epidemiologic studies. As an example, in vitro studies on cancer demonstrated that apples and apple juice could exert chemoprotective effects through alteration of carcinogen metabolism, antioxidant and anti-inflammatory capacity, inhibition of proliferation, apoptosis, alteration of signaling pathways, epigenetic modifications, antimutagenic activity, innate immunity and others [90]. Koch et al. [91] investigated the potential mechanisms underlying the associations found between apple juice consumption and colorectal cancer risk in epidemiologic studies focusing on in vitro studies, and gathered a wide variety of plausible mechanisms. These mechanisms included, among others, antioxidant activity, inhibition of cell proliferation, modulation of gene expression, onset of apoptosis, improvement of immune system, and alteration of signaling pathways (such as those mentioned above), but also an increase in enzymatic activity implicated in cell response to toxic substances and a decrease in enzyme activity related to oxidoreduction [91]. Moreover, Ortiz and Shea showed that apple juice prevented the generation of reactive oxygen species, the increase in cytosolic calcium and apoptosis induced by cell exposure to beta-amyloid in a dose-response pattern [92]. Since beta-amyloid is closely related to Alzheimer’s disease, this last result reinforces the hypothesis that apple juice consumption could exert neuroprotective activity [92]. In another in vitro study, it was demonstrated that apple juice inhibited copper catalyzed human LDL oxidation [93]. Overall, despite that relevant several clinical studies complemented results from epidemiological and in vitro studies, several parameters, markers and diseases still remain to be studied. In addition, the health impact of pesticides should be further investigated. Current literature on the subjects appears to be inconsistent. Although pesticide residues can be found in apples [94], the current data tends to support that they do now represent a major threat to human health [95,96]. Clarification of the impact of pesticides on health is therefore still needed.

In conclusion, despite that all mechanisms by which apple juice exerts its biological activities to prevent the development of chronic diseases are yet to be fully understood, studies overall suggest that apple juice consumption has positive effects on markers related to cardiovascular disease, cancer, neurodegenerative diseases, and probably others. The beneficial effects of apple juice are more apparent with cloudy apple juice than clear apple juice, probably due to its lower nutritional density. It is likely that whole apples are superior to apple juice. However, apple juice may constitute a convenient and easy way to increase fruit consumption and improve overall quality of the diet when consumed in moderation.

## Figures and Tables

**Table 1:** Summary of all interventional trials included in the present review.

Authors	Design	Subjects	Purpose	Intervention	Main Results
Barth SW 2012	Controlled, randomized, and parallel study.	68, non-smoking, non-diabetic men with a BMI ≥ 27 kg/m^2^.	To verify the effect of polyphenol-rich cloudy apple juice (CloA) consumption on plasma parameters related to the obesity phenotype and potential effects of interactions between CloA and allelic variants in obesity candidate genes in obese men.	Consumption of 750 mL/day of polyphenol-rich CloA (802.5 mg polyphenols) or 750 mL/day control beverage (CB, isocaloric equivalent to CloA) for 4 weeks.	CloA compared to CB had no significant effect on plasma lipids, plasma adipokine and cytokine levels, BMI, and waist circumference. CloA consumption significantly reduced percent body fat compared to CB.
Erickson J 2017	Double-blind, randomized, controlled crossover study.	40 healthy adults.	To determine whether there is a difference in gastro-intestinal (GI) tolerance between juice from a high-FODMAP fruit (apple juice) and juice from a low-FODMAP fruit (white grape juice) in healthy human subjects in order to provide insight into the role of juice in a low-FODMAP diet.	Fasted subjects consumed 12 oz of either apple juice or white grape juice. Measures of breath hydrogen were taken at baseline, 1, 2, and 3 h. Subjective GI tolerance surveys were completed at the same time intervals and at 12 and 24 h.	Consumption of apple juice resulted in a greater mean breath hydrogen area under the curve at 23.3 ppm/hour compared with white grape juice at 5.8 ppm/hour (*p* < 0.001). No differences in reported GI symptoms were seen between treatments.
Godycki- Cwirko M 2010	Double-blind placebo-controlled design.	12 healthy nonsmoking subjects.	To determine whether (1) rapid consumption of 1 L of apple juice increases blood antioxidant capacity, measured FRAP and DPPH radical-scavenging activity, and (2) apple polyphenols or fructose-induced elevation of plasma uric acid contributes to post-juice increase of blood antioxidant activity.	Subjects consumed 1 L of clear apple juice and then FRAP; serum DPPH scavenging activity, serum uric acid, and total plasma phenolics and quercetin levels were measured just before juice ingestion and 1, 2.5, and 4 h after ingestion. This was repeated 3 times with 4-day intervals, but volunteers drank either 1 L of clear apple juice without polyphenols (placebo), or 1 L of cloudy apple juice (positive control), or 1 L of water (negative control) at the time.	Consumption of all 3 juices transiently increased FRAP and serum DPPH-scavenging activity, with peak values at 1 h post-juice ingestion. This was paralleled by the rise of serum uric acid, but no significant changes in plasma total phenolics and quercetin levels were observed after all dietary interventions. At the same time, no substantial differences were found between juices (especially between clear apple juice and clear apple juice without polyphenols) concerning the measured variables.
Guo C 2008	Randomized without a control group.	26 nonsmokers subjects elderly (older than 60 years old): 20 men and 6 women.	To compare the efficacy of pomegranate juice and apple juice in improving antioxidant function in elderly subjects.	2 groups, that is, apple (low in antioxidant capacity) and pomegranate (high in antioxidant capacity) groups, and 250 mL of juice was consumed daily for 4 weeks.	Increased plasma antioxidant capacity and decreased plasma carbonyl content were demonstrated after daily consumption of pomegranate juice. In comparison, apple juice consumption presented a less significant effect on antioxidant function in elderly subjects.
Hagl S 2011	Unspecified	Ten healthy ileostomy subjects aged between 39 and 72 years.	The aim of this study was to determine the amounts of polyphenols and quinic acid reaching the ileostomy bags of probands (and thus the colon in healthy humans) after ingestion of apple smoothie, a beverage containing 60% cloudy apple juice and 40% apple puree.	Ingestion of 0.7 L of apple smoothie (a bottle). Their ileostomy bags were collected directly before and 1, 2, 4, 6 and 8 h after smoothie consumption, and the polyphenol and quinic acid contents of the ileostomy fluids were examined.	The amounts of polyphenol and quinic acids reaching the ileostomy bags are considerably higher after apple smoothie consumption than after the consumption of cloudy apple juice or cider. These results suggest that the food matrix might affect the colonic availability of polyphenols, and apple smoothies could be more effective in the prevention of chronic colon diseases than both cloudy apple juice and apple cider.
Hyson D 2000	Unblinded, randomized, crossover design.	12 healthy men and 13 healthy women.	To examine the in vivo effect of consumption of apples (both whole and juice).	The addition of 375 mL of unsupplemented apple juice or 340 g of cored whole apple to their daily diet for 6 weeks, then crossed over to the alternate product for 6 weeks. Blood samples were obtained at baseline and after each dietary period.	Apple juice consumption increased ex vivo copper (Cu ^+ +^)-mediated LDL oxidation lag time by 20% compared with baseline. Apples and apple juice both reduced conjugated diene formation. Moderate apple juice consumption provides in vivo antioxidant activity. In view of the current understanding of CAD, the observed effect on LDL might be associated with reduced CAD risk and supports the inclusion of apple juice in a healthy human diet.
Kahle K 2007	Unspecified	11 healthy ileostomy subjects.	To characterize new metabolites of hydroxycinnamic acids and dehydrochalcones in the ileostomy effluent.	Subjects consumed 1 L of cloudy apple juice. Ileostomy bags were removed 0, 1, 2, 4, 6, and 8 h after juice consumption.	Ninety percent of the consumed procyanidins were recovered in the ileostomy effluent and therefore would reach the colon under physiologic circumstances. The gastrointestinal passage seems to play an important role in the colonic availability of apple polyphenols.
Ko SH 2005	Unspecified	10 healthy men 25–26 years old with an average body mass index of 21.8 ± 2.3 kg/m^2^.	To test the hypothesis that the consumption of fruit juices may improve antioxidant status in human plasma.	After overnight fasting, study subjects were fed 150 mL of fruit juice, and blood was collected at 0, 30, 60, 90, and 120 min after consumption. After a 1-day wash-out period, subjects were fed with the next sample of fruit juice until all nine juices (pear, apple, orange, grape, peach, plum, kiwi, melon, and watermelon) had been evaluated.	Except for pear juice, eight kinds of juices exhibited potent antioxidant effects in human plasma. Within 30 min after consumption, orange, melon, grape, peach, plum, apple, and kiwi juices already effectively suppressed reactive oxygen species generation. his radical scavenging effect of fruit juices was maintained for up to 90 min post-consumption. These results suggest that the consumption of fruits or fruit juices may reduce damage from oxidative stress, and that this effect may be a consequence of the antioxidant activity of fruits in scavenging the reactive oxygen species generated in human plasma.
Ravn-Haren G 2013	Crossover study.	23 healthy volunteers.	To assess the effects of whole apples (550 g/day), apple pomace (22 g/day), clear and cloudy apple juices (500 mL/day), or no supplement on lipoproteins and blood pressure	5 × 4 weeks dietary crossover study to assess the effects of whole apples (550 g/day), apple pomace (22 g/day), clear and cloudy apple juices (500 mL/day), or no supplement.	Trends towards a lower serum LDL concentration were observed after whole apple (6.7%), pomace (7.9%) and cloudy juice (2.2%) intake. On the other hand, LDL-cholesterol concentrations increased by 6.9% with clear juice compared to whole apples and pomace.
Remington R 2010	Open-label clinical trial.	21 institutionalized individuals with moderate to severe Alzheimer’s disease (AD).	To verify the efficacy of apple juice in cognition and mood in AD.	Consumption of 2,4-oz glasses of apple juice daily for 1 month.	Participants demonstrated no change in the Dementia Rating Scale, and institutional caregivers reported no change in Alzheimer’s Disease Cooperative Study (ADCS)-Activities of Daily Living (ADL). This pilot study suggests that apple juice may be a useful supplement, perhaps to augment pharmacological approaches, for attenuating the decline in mood that accompanies progression of AD, which may also reduce caregiver burden.
Shah M 2003	Random order of juice consumption.	25 healthy children, 3 to 6 years of age.	To measure iron absorption in children from meals containing apple juice or orange juice so as to determine if iron absorption will be greater with orange juice due to its higher ascorbic acid content than apple juice.	On 2 successive days, children consumed identical meals that included apple juice on one day and orange juice on the other. Iron absorption was measured from red blood cell incorporation of the iron stable isotopes 14 days later.	Median iron absorption from the meal ingested with apple juice was 7.17% while it was 7.78% with orange juice (*p* = 0.44).
Soriano-Maldonado 2014	Randomized crossover study.	20 subjects, aged 21–29 years.	To investigate the effects of the consumption of two cloudy apple juices with different polyphenol and vitamin C contents on antioxidant status, cardiometabolic and inflammation markers in healthy young adults.	At each 4-week intervention period, the volunteers randomly consumed two glasses (2 × 250 mL/day) of either a vitamin C-rich apple juice (VCR) (60 mg/L vitamin C and 510 mg catechin equivalent/L) or a polyphenol-rich (PR) juice (22 mg/L vitamin C and 993 mg catechin equivalent/L).	During the VCR period, plasma antioxidant activity (FRAP) increased (*p* = 0.031), while ICAM-1 and total cholesterol showed a trend to decrease. During the PR period, plasma insulin and HOMA increased, and total glutathione decreased (*p* < 0.05). A joint consumption of apple juice natural antioxidants such as vitamin C and polyphenols might provide mild favorable effects on cardiometabolic markers, as compared to apple polyphenols alone.
Trost K 2018	Randomized crossover study.	12 men and women (8 males and 4 females), aged 21 to 42 years, with a BMI between 18.5 and 25 kg/m^2^ (normal weight).	To investigate the nutrikinetics of apple polyphenols by UHPLC-HRMS metabolite fingerprinting, comparing bioavailability when consumed in a natural or a polyphenol-enriched cloudy apple juice.	Consumption of 250 mL of cloudy apple juice (CAJ), Crispy Pink apple variety, or 250 mL of the same juice enriched with 750 mg of an apple polyphenol extract (PAJ). Plasma and whole blood were collected at time 0, 1, 2, 3 and 5 h. Urine was collected at time 0 and 0–2, 2–5, 5–8, and 8–24 h after juice consumption. Faecal samples were collected from each individual during the study for 16S rRNA gene profiling.	As many as 110 metabolites were significantly elevated following intake of polyphenol enriched cloudy apple juice, with large inter-individual variations. The comparison of the average area under the curve of circulating metabolites in plasma and in urine of volunteers consuming either the CAJ or the PAJ demonstrated a stable metabotype, suggesting that an increase in polyphenol concentration in fruit does not limit their bioavailability upon ingestion.
Veeriah S 2008	Unspecified	11 volunteers.	To assess related mechanisms caused by ileostomy samples from volunteers that had consumed apple juice.	Ileostomy samples were collected after intervention (0–8 h) with cloudy apple juice (1 L). All volunteers drank 1 L of cloudy apple juice within 15 min. A light meal which did not contain polyphenols was served 4 h later. The ileostomy bag was removed before (control value) and 1, 2, 4 and 6 h after the start of the apple juice intake.	The analytical determination of polyphenols in the ileostomy samples revealed that the majority of the compounds were recovered in the samples collected 2 h after intervention. The intervention with apple juice results in bioavailable concentrations of related polyphenols in the gut lumen, which could contribute to reduced genotoxicity, enhanced antigenotoxicity and favorable modulation of GSTT2 gene expression in some individuals.
Vieira FG 2012	Randomized crossover study.	9 healthy women.	To determine the antioxidant capacity and the levels of ascorbic and uric acids, total phenols, lipid hydroperoxides (LH), and thiobarbituric acid-reactive substances (TBARS) in the serum of 9 healthy individuals 1 h after the intake of Golden Delicious or Catarina AJ.	300 mL of Golden Delicious or Catarina apple juice (AJ) or water, and blood samples were collected before and 1 h after intake.	After intake of both AJ, a similar and significant increase in serum antioxidant capacity and ascorbic and uric acid levels and a significant decrease in serum lipid peroxidation was observed. The increase in serum antioxidant capacity after consumption of both AJ was correlated directly with the uric acid levels and inversely with serum lipid peroxidation.
White SJ 2018	Randomized crossover study.	73 participants (58 women, 15 men).	To test whether fructose present in fruit is of sufficient quantity or in a form that will increase uric acid concentration.	Three groups to ingest small (205 g) and large (410 g) servings of apple segments, small (170 mL) and large (340 mL) servings of apple juice, or a glucose and a fructose control beverage. The fructose control and the large servings of apple and juice contained 26.7 g fructose. Test foods were ingested within 10 min. Blood samples were taken at baseline and at 30 and 60 min after intake.	The mean increase in uric acid at 30 min was 15 μmol/L (10, 21 μmol/L) for the fructose control and 19 μmol/L (8, 30 μmol/L) and 17 μmol/L (9, 24 μmol/L) for the large servings of apple and apple juice, respectively. There was no difference in change in uric acid between baseline and 30 min when comparing the apple and apple juice with the fructose control. Blood pressure taken 70 min after ingestion was unaffected by any treatment (*p* > 0.05). There was no difference in change in satiety scores between the fructose and glucose control beverages (*p* > 0.05).
Wruss J 2015	Unspecified	35 healthy students of normal weight split equally across the two study sites (pool A: 17; pool B: 18) and gender (20 female, 15 male) aged between 19 and 42.	To determine the pharmacokinetic fate of apple polyphenols in young healthy adults.	Volunteers consumed 500 mL of an unfiltered apple juice. Blood and urine samples were collected within a time period of ten hours and analyzed for their total phenolic content.	An increase in the total phenolic content over time did not correlate with an observed, highly elevated antioxidant capacity (AOC) in the blood plasma, which was rather a result of a high fructose content of the apple juice.
Yuan L 2011	Unspecified	26 healthy young subjects (13 male and 13 female aged 20–23 years).	To investigate the influences of apple and grape juices consumption on body antioxidant status.	Each subject received 100% purified fruit juice twice a day (300 mL apple juice at lunch and 300 mL grape juice at dinner) for 2 weeks. Fasting venous blood samples were collected before and after 2 weeks of intervention from each subject.	Apple and grape juice consumption increased the plasma T-AOC and decreased the concentration of malondialdehyde. Erythrocyte glutathione peroxidase and catalase activities were enhanced. No effect was observed in plasma carbonyl content, lymphocyte damage or urinary 8-hydroxy-2-deoxyguanosine. These findings indicated that concomitant intake of apple and grape juice was efficient in enhancing the body’s antioxidant status.

## Data Availability

This study did not report any new data.

## Supplementary Materials

The following supporting information can be downloaded at: https://www.mdpi.com/article/10.3390/nu14040821/s1, Table S1: Dietary value of a medium-sized apple with skin; Table S2: Dietary value of a clarified apple juice (in can or bottle).
