# Supplementary material for: Health Benefits of Apple Juice Consumption: A Review of Interventional Trials on Humans
Source: Nutrients. 2022 Feb 16;14(4):821. doi: 10.3390/nu14040821 (PMC8879758; doi:10.3390/nu14040821)
Supplement: Supplementary file 1 [file nutrients-14-00821-s001.zip › nutrients-1545821-supplementary.pdf]

nutrients MDPI

Supplementary Material

Table S1. Dietary value of a medium-sized apple with skin.

Nutrient	Unit	1 medium fruit (7cm diameter/182 g)
**Macronutrients**		
Moisture	g	155.72
Ash	g	0.35
Protein	g	0.47
Total Fat	g	0.31
Carbohydrate	g	25.13
Energy	kCal	95
**Carbohydrates**		
Fibre, total dietary	g	3.5
Starch	g	0.09
Glucose	g	4.42
Fructose	g	10.74
Maltose	g	0.00
Galactose	g	0.00
Lactose	g	0.00
Sucrose	g	3.77
Sorbitol	g	0.55
Sugars, total	g	18.91
**Minerals**		
Calcium, Ca	mg	11
Iron, Fe	mg	0.22
Magnesium, Mg	mg	9
Phosphorus, P	mg	20
Potassium, K	mg	195
Sodium, Na	mg	2
Zinc, Zn	mg	0.07
Copper, Cu	mg	0.049
Manganese, Mn	mg	0.064
Selenium, Se	µg	0.0
**Vitamins**		
Beta carotene	µg	49
Alpha carotene	µg	0
Retinol	µg	0
Retinol activity equivalents, RAE	µg	5
Folacin, total	µg	5

Folic acid, synthetic form	μg	0
Folate, naturally occurring	μg	5
Dietary folate equivalents, DFE	μg	5
Niacin	mg	0.166
Niacin equivalents	NE	0.196
Pantothenic acid	mg	0.111
Riboflavin	mg	0.047
Thiamin	mg	0.031
Vitamin B-6	mg	0.075
Vitamin B-12	μg	0.00
Choline, total	mg	6.2
Betaine	mg	0.2
Vitamin C	mg	8.4
Vitamin D	μg	0.0
Vitamin D (IU)	IU	0
Vitamin K	μg	4.0
Tocopherol, alpha	mg	0.33
Tocopherol, beta	mg	0.00
Tocopherol, delta	mg	0.00
Tocopherol, gamma	mg	0.00
**Amino Acids**		
Tryptophan	g	0.002
Threonine	g	0.011
Isoleucine	g	0.011
Leucine	g	0.024
Lysine	g	0.022
Methionine	g	0.002
Cystine	g	0.002
Phenylalanine	g	0.011
Tyrosine	g	0.002
Valine	g	0.022
Arginine	g	0.011
Histidine	g	0.009
Alanine	g	0.020
Aspartic acid	g	0.127
Glutamic acid	g	0.046
Glycine	g	0.016
Proline	g	0.011
Serine	g	0.018
**Lipids**		
Fatty acids, saturated, total	g	0.051
Fatty acids, monounsaturated, total	g	0.013

Fatty acids, polyunsaturated, total	g	0.093
Total plant sterol	mg	22
Cholesterol	mg	0
**Other components**		
Caffeine	mg	0
Theobromine	mg	0
Lycopene	µg	0
Beta cryptozanthin	µg	20

Whereas Phytochemicals taken from the Canadian Nutrient File (7).

Table S2. Dietary value of a clarified apple juice (in can or bottle).

Nutrient	Unit	Value per 250ml (portion)
**Macronutrients**		
Moisture	g	231.23
Ash	g	0.60
Protein	g	0.26
Total Fat	g	0.34
Carbohydrate	g	29.61
Energy	kCal	121
**Carbohydrates**		
Fibre, total dietary	g	0.5
Glucose	g	6.89
Fructose	g	15.02
Maltose	g	0.00
Galactose	g	0.00
Lactose	g	0.00
Sucrose	g	3.30
Sorbitol	g	2.62
Sugars, total	g	25.21
**Minerals**		
Calcium, Ca	mg	21
Iron, Fe	mg	0.31
Magnesium, Mg	mg	13
Phosphorus, P	mg	18
Potassium, K	mg	265
Sodium, Na	mg	10
Zinc, Zn	mg	0.05
Copper, Cu	mg	0.031
Manganese, Mn	mg	0.194
Selenium, Se	µg	0.3
**Vitamins**		
Beta carotene	µg	0
Retinol	µg	0
Retinol activity equivalents, RAE	µg	0
Folacin, total	µg	0
Folic acid, synthetic form	µg	0
Folate, naturally occurring	µg	0
Dietary folate equivalents, DFE	µg	0
Niacin	mg	0.191
Niacin equivalents	NE	0.239
Pantothenic acid	mg	0.128

Riboflavin	mg	0.045
Thiamin	mg	0.055
Vitamin B-6	mg	0.047
Vitamin B-12	µg	0.00
Choline, total	mg	4.7
Betaine	mg	0.3
Vitamin C	mg	2.4
Vitamin D	µg	0.0
Vitamin D (IU)	IU	0
Vitamin K	µg	0.0
Tocopherol, alpha	mg	0.03
**Lipids**		
Fatty acids, saturated, total	g	0.058
Fatty acids, monounsaturated, total	g	0.016
Fatty acids, polyunsaturated, total	g	0.102
Cholesterol	mg	0
**Other components**		
Caffeine	mg	0
Theobromine	mg	0
Phenolic compounds* (hydroxycinnamic acids and flavonoids)	mg	62.64* (in comparison in apple mash: 114.8*)
